# The Plasticizing Effect of Choline Dihydrogencitrate on Starch-Based Films

**DOI:** 10.3390/ma19153242

**Published:** 2026-07-31

**Authors:** Dorota Skowrońska, Katarzyna Wilpiszewska

**Affiliations:** Department of Chemical Organic Technology and Polymeric Materials, Faculty of Chemical Technology and Engineering, West Pomeranian University of Technology, Pułaskiego 10, 70-322 Szczecin, Poland; dorota.skowronska@zut.edu.pl

**Keywords:** choline dihydrogencitrate, potato starch, plasticization

## Abstract

For the first time, choline dihydrogencitrate salt (CCT) was tested as a sole plasticizer for starch-based materials. The effect of CCT content on the physicochemical properties of the obtained films was evaluated. The FTIR and XRD measurements revealed that CCT exhibited the ability to disrupt the crystalline regions of potato starch. Also, DMTA and the mechanical test results showed that CCT acted as a primary plasticizer, leading to increased flexibility of starch-based films. Considering the transparency and mechanical properties, the efficient content of CCT was 25 wt.%. The obtained materials exhibited lower moisture absorption levels when compared to other common starch plasticizers, which makes them interesting as less moisture-sensitive films for packaging.

## 1. Introduction

Large amounts of plastic waste is a growing worldwide problem [1]. The majority of the packaging materials are produced from petroleum-based thermoplastics. Replacing at least a part of such materials with biodegradable polymers could limit this issue. Moreover, using natural polymers contributes to the reduction of carbon emissions [2].

Starch is a widely available, biodegradable, and carbon-neutral biopolymer. The products of starch degradation are water and carbon dioxide; thus, using this polysaccharide follows the principles of a circular economy [3,4]. Starch is used for many non-food applications, as an adhesive, binding agent, or filler, in the paper, pharmaceutical, and textile industries. Starch consists of linear amylose and branched amylopectin, and the amylose/amylopectin ratio depends on the botanical origin. By thermomechanical treatment of starch in the presence of a plasticizer, thermoplastic starch can be formed. Generally, the presence of plasticizers results in a disordered starch phase and a reduction in the polymer glass transition temperature and viscosity [5,6]. The most popular starch plasticizer is water and other low-molecular-weight substances containing OH or NH_2_ groups (e.g., glycerol, ethylene glycol, urea) [7]. However, the efficiency of plasticization depends on the plasticizer type and content. Moreover, an efficient plasticizer should be compatible with the polysaccharide for its homogeneous distribution in the polysaccharide matrix [8]. The plasticizer can interact with polysaccharide molecules (i) by replacing hydrogen bonds between starch OH groups with starch-plasticizer bonds, or (ii) by acting as an agent that attracts and holds a large number of water molecules, plasticizing the polysaccharide [8]. This is the reason why the properties of thermoplastic starch also depend on the moisture content. 

Water is the most basic starch plasticizer; however, it is volatile and easily evaporates during processing. Other common low-molecular-weight plasticizers, after some time, tend to diffuse out of the polysaccharide matrix, making the properties of the final material unstable [9]. 

In the last decade, ionic liquids (ILs) were tested for starch dissolution and plasticization. The most common ILs for that purpose were those based on imidazolium cations [10]; however, they are considered harmful to the environment [11,12], and their toxicity increased with the number of nitrogen atoms in the cation ring [13]. More environmentally friendly ILs are based on cholinium cation and salts of organic acids [11]. Choline chloride is the precursor of vitamin B4, biocompatible, and used for animal feed [14,15]. The choline ionic liquids (choline derivatives: lactate, acetate, and chloride) were used for the plasticization of corn starch [15]. Importantly, it was reported that neat choline chloride cannot plasticize dry starch [16]. However, choline chloride in the presence of water led to a similar decrease in glass transition temperature of starch as its conventional plasticizers, i.e., glycerol [15].

Choline dihydrogencitrate salt (CCT) has been used for over 80 years in human medicine [17] and for animal feed [18]. It can be classified as an environmentally friendly ionic liquid. CCT was recently used as a hydrogen bond-accepting component of deep eutectic solvent (DES) applied for plasticizing starch [19,20]. As the hydrogen bond donors, glycerol and urea were used. DES obtained from CCT and glycerol or urea exhibited the ability to disrupt the crystalline arrays of starch [20]. Moreover, a ternary DES system CCT/glycerol/urea was tested [21,22]. With increasing the ternary plasticizer content (from 20 phr up to 50 phr), the tensile strength of starch-based films decreased, and elongation at break increased. Interestingly, besides plasticizing activity, the CCT-based eutectic solvents also acted as a crosslinking agent [19,20,21].

We hypothesize that choline dihydrogencitrate salt, considered as an ionic liquid, could exhibit plasticizing activity toward starch (independently, not as a component of the DES system). For this purpose, the starch/choline dihydrogencitrate salt films were prepared with various CCT contents, and their effects on morphology, moisture absorption, as well as thermal and mechanical properties were evaluated.

## 2. Materials and Methods

### 2.1. Materials

**Figure 1:**
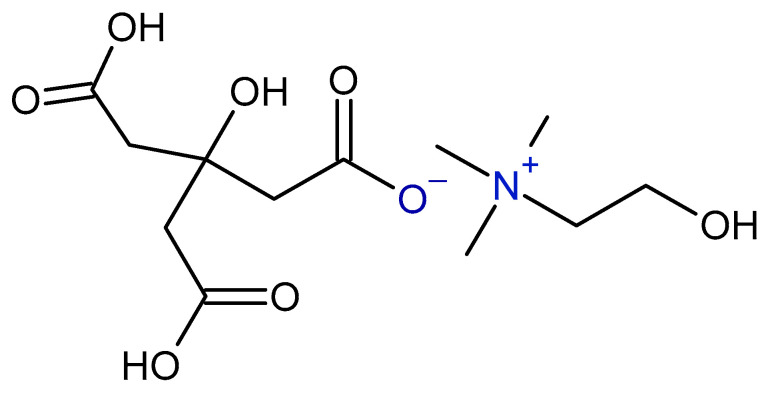
The scheme of choline dihydrogencitrate salt structure.

Potato starch (S, containing 16% moisture, 0.06% proteins, and 0.3% ash content; amylose/amylopectin ratio 26/74 wt.%) was a product of Zetpezet Piła (Poland). Choline dihydrogencitrate salt (CCT, ≥98%) was purchased from Sigma-Aldrich (Figure 1).

### 2.2. Methods

#### 2.2.1. Preparation of Starch Films Plasticized with Choline Dihydrogencitrate Salt

The starch (3 g) was suspended in 100 g distilled water and heated up to 90 °C for 30 min while stirring. After starch gelatinization, the CCT was added (0, 10, 20, 25, 30, or 35 wt.% based on dry starch; the samples were labeled according to their CCT content, e.g., S_CCT_30) and mixed at room temperature until homogeneous. The obtained solution was poured into the Petri dish and dried at 60 °C.

#### 2.2.2. Viscosity of the Film-Forming Systems

The Brookfield viscometer, model RVT (Brookfield Engineering Laboratories), was applied to determine the viscosity of the aqueous starch/CCT film-forming solutions (60 °C). First, starch was gelatinized in water (3 wt.%), and the effect of various CCT (10−35 wt.%) contents was determined. The samples were labeled according to their CCT content, e.g., S_SCCT_30.

#### 2.2.3. Fourier Transform Infrared Spectroscopy (FTIR)

The FTIR analyses of the films were performed in a Nexus FTIR Spectrometer Thermo Nicolet with Golden Gate ATR mode (Waltham, MA, USA). For each starch-based sample, 32 scans were taken from 4000 to 400 cm^−1^. The resulting spectra were converted using the software OMNIC v.32.

#### 2.2.4. X-Ray Diffractometry (XRD)

The crystallinity of starch films was analyzed by X-ray diffraction (X’Pert Pro, PANalytical, Malvern Panalytical Ltd., Malvern, UK; operated at the CuK(alfa) wavelength 1.54 Å).

#### 2.2.5. Differential Scanning Calorimetry (DSC)

Phase transitions in starch/CCT systems were investigated using the DSC technique (Q100, TA Instruments, New Castle, DE, USA) after 24 h of storage. The aluminum hermetic pans (ca. 10 mg of the sample) with nitrogen as a cooling agent, with a heating rate of 5 °C/min, were applied. A standard run at the temperature range from −80 to 200 °C was performed. Additionally, in similar conditions, the measurements of the powder mixture of starch and CCT were performed.

#### 2.2.6. Thermogravimetric Analysis (TGA)

The thermal stability of starch/CCT films was investigated using TGA (Q500, TA Instruments, New Castle, DE, USA). Tests were performed on platinum pans under 25 mL/min airflow, in the temperature range of 40–700°C at a heating rate of 10 °C/min.

#### 2.2.7. Mechanical Properties

Mechanical properties were measured using a tensile tester (Instron 4026, Instron Corporation, Marwood, MA, USA) equipped with a 1 kN load cell. The film specimens (10 mm × 100 mm strips) were conditioned at RH = 55% for 24 h before tests. The initial grip separation and cross-head speed were 50 mm and 10 mm/min, respectively. The mechanical tensile data were averaged over at least six specimens.

#### 2.2.8. Moisture Absorption

The moisture absorption tests were performed for the starch-based films. For each film, three squares (1.5 cm^2^) were prepared and placed in a desiccator for two weeks to dry. Dry samples were weighed and subsequently placed in a climatic chamber (55 ± 2% humidity, 25 ± 2 °C). The tested samples were weighed at 1, 3, 6, 24, 48, 72, and 120 h after placing the samples in a climatic chamber. Moisture absorption was calculated using Equation (1) [23]:

Moisture absorption (%) = [(m_t_ − m_0_)/m_0_] × 100 (1)

where: m_0_—mass of dry sample (g), and m_t_—mass of sample after time t (g).

## 3. Results and Discussion

**Figure 2:**
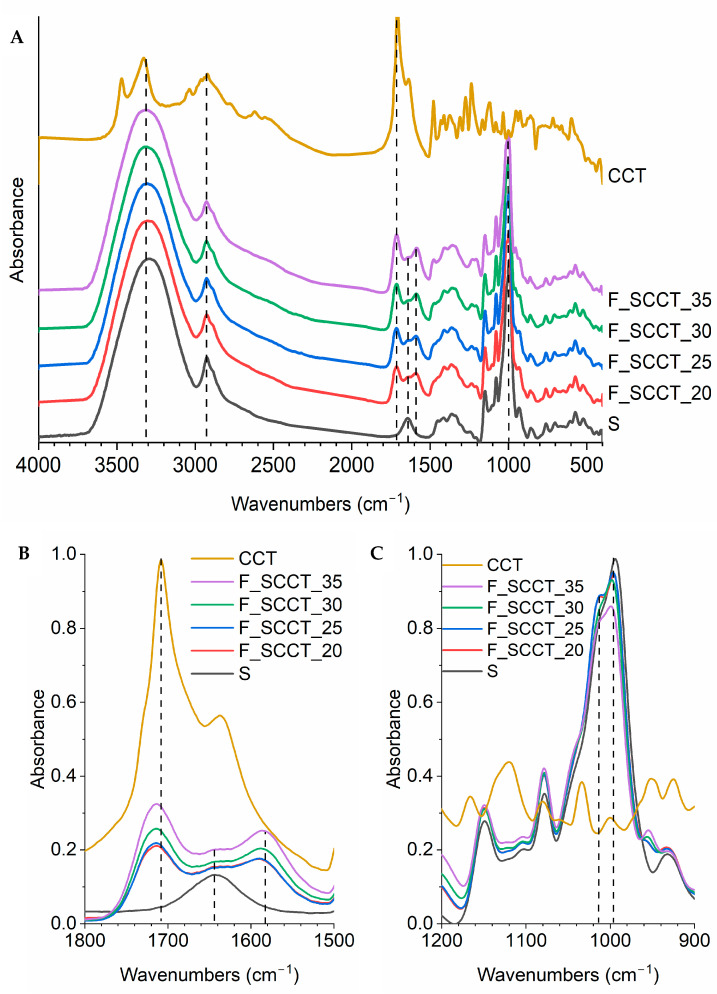
The FTIR spectra of CCT and starch-based films with various CCT contents in (**A**) the full wavenumber range, (**B**) the wavenumber range of 1500–1800 cm^−1^, and (**C**) the wavenumber range of 900–1200 cm^−1^.

The FTIR spectra of the starch films with various CCT contents were collected in Figure 2. The FTIR spectra for the systems with low CCT was presented in Figure S1. The broad absorption band in the range 3000–3500 cm^−1^, attributed to stretching vibrations of hydroxyl groups of starch, was observed for all the films. With increasing CCT content, the intensity of this band slightly decreased, indicating the formation of intermolecular hydrogen bonds between the hydroxyl groups of starch and CCT. Owing to the multifunctional structure of CCT, it is capable of creating a multi-dentate hydrogen-bonding network, in which several hydroxyl and carboxyl groups simultaneously interact with neighboring starch chains. Such interactions partially replace starch–starch hydrogen bonds with starch–CCT hydrogen bonds, leading to changes in the local molecular environment. The characteristic C-H stretching at ca. 2950 cm^−1^ [24] and fingerprint pattern of native starch in the range 900–1200 cm^−1^, assigned to stretching C-O and C–C as well as bending C-O–H groups from polysaccharide units [25], remained present in all systems. The broad band typical for starch at ca. 1640 cm^−1^ (Figure 2B), assigned to O-H bending [24], was slightly noticeable for starch/CCT systems. However, a new band at ca. 1715 cm^−1^, corresponding to bending C=O from protonated carboxylic groups of CCT, appeared. The intensity of a carbonyl band correlated with choline citrate content. This observation confirms the increasing contribution of citrate-derived functional groups to the hydrogen-bonding network.

Interestingly, the absorption band in the CCT spectrum at ca. 1635 cm^−1^, attributed to carboxylate salt, shifted to a lower wavenumber (ca. 1580 cm^−1^) in the spectra of starch/CCT systems [26], and its intensity correlated with CCT content. This reveals stronger intermolecular interactions involving carboxylate groups and indicates that these groups participate in hydrogen bonding with starch hydroxyls.

The band at 994 cm^−1^ is sensitive to starch-plasticizer interactions involving hydrogen bonds (Figure 2C). Changes in the intensity of this band correlate with changes in the polysaccharide primary hydroxyl groups. Furthermore, the band at ca. 1040 cm^−1^ was ascribed to starch crystallinity—intensity decrease indicates an increase in the amorphous phase content [27]. As the intensity bands decreased with CCT content, it could be concluded that the disruption of the native starch hydrogen-bonding network by the interactions with CCT leads to a more amorphous structure of polysaccharide. 

Due to its multifunctional structure, CCT has a greater potential to form an extended hydrogen-bonding network within the starch matrix than conventional starch plasticizers like glycerol.

**Figure 3:**
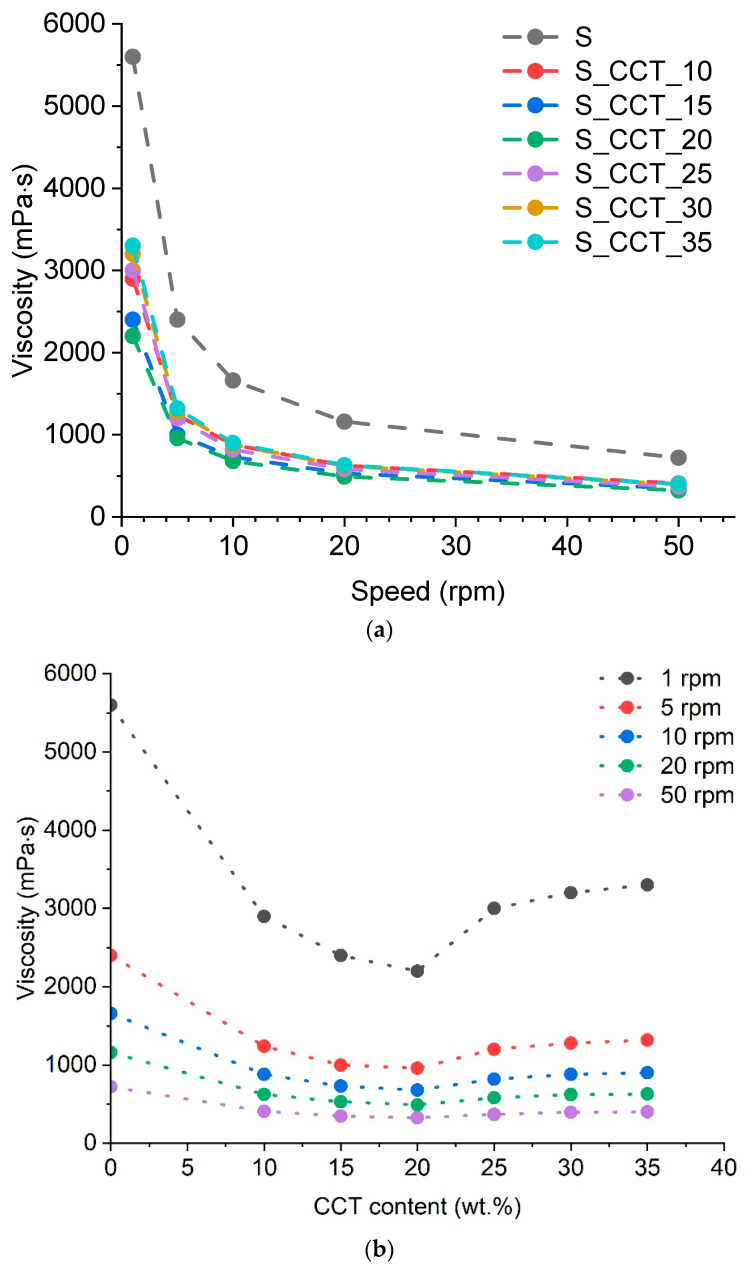
The viscosity of starch/CCT systems as a function of (**a**) rotational speed and (**b**) CCT content.

The viscosity of the aqueous starch solutions containing various CCT contents was tested for shear rates in the range 1 to 50 rpm (Figure 3). The systems were non-Newtonian liquids exhibiting shear-thinning behavior (the viscosity drop with shear rate increase), indicating that the polymer network had a notable change (Figure 3a). Additionally, the viscosity for the neat starch solution was even higher than the system containing the lowest CCT dosage (10 wt.%). This means that the presence of CCT weakened the intermolecular forces between polysaccharide chains [28]. A similar effect was observed for the glycerol and PEG-plasticized chitosan/zein film-forming solutions [29]. Considering the effect of CCT content on the viscosity value, interestingly, a minimum on the viscosity curve could be observed for 20 wt.% CCT content (Figure 3b). Such a viscosity drop was noted for each shear rate value. According to the mechanistic theory of plasticization by Doolittle [28], the plasticizer molecules are constantly attracted, but none of the plasticizer molecules is permanently bonded to a macromolecular chain. Instead, the attached plasticizer is dynamically replaced by another. Thus, no stoichiometric ratio between the polymer and plasticizer could occur. However, there are some empirical examples that allow for anticipation of the quantitative relationship between the polymer matrix and plasticizer for PVC plasticization [30]. They were conducted mostly for PVC, as the plasticization technology started with this polymer. A similar assumption regarding starch plasticization will be subject to greater error because starch is a mixture of two polymers. However, despite the inherent limitations associated with adapting an equation originally developed for PVC systems to starch-based materials, an exploratory analysis was undertaken to evaluate its applicability. This approach was used only as an approximate method for estimating the plasticizer content, without assuming that the plasticization mechanism of starch is identical to that of PVC. Accordingly, the plasticizer amount (Pl) required theoretically to interact with one active group in each repeating unit of the starch chain could be determined as:

Pl (phr) = (M × 100)/972(2)

where M is the molecular weight of the plasticizer, and 972 is the molecular weight of the helical unit of amylose.

In the case of CCT, the theoretically efficient amount is 30 phr (i.e., ~30 wt.%). However, the viscosity results revealed that the CCT content of 20 wt.% was the most efficient in penetrating the starch structure in the aqueous system. There is an excess of water in the tested systems; it could be concluded that water enhanced the plasticizing effect of CCT.

**Figure 4:**
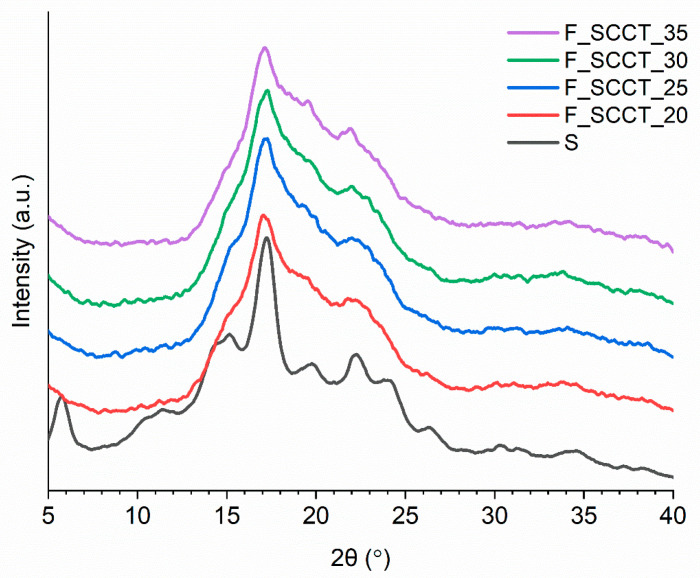
XRD patterns of native starch (S) and starch-based films with various CCT contents.

In Figure 4, the XRD patterns of starch-based films are presented. Due to the brittleness, only the films containing at least 20 wt.% plasticizer were selected for further tests. Amylose and amylopectin form semicrystalline arrays in the starch granules [31]. The crystallinity measurements allow for determining the presence of long-range crystalline order and could give an insight into the plasticization activity [32]. Native potato starch exhibits a B-type crystalline polymorphic form, which is common in tubers [33]. The characteristic diffraction peaks at ca. 5, 11, 15, 17, 19, 22, 24, and 26° [33] for native potato starch could be observed (Figure 4). For the systems containing choline ionic liquid, the signals at 5, 11, 15, 19, 24, and 26° disappeared, and those at 17 and 22° became less intense, indicating a significant increase in the amorphous phase [20]. The XRD results correlate with the conclusions from FTIR spectra analysis.

Lowering the crystallinity suggested that CCT could disrupt the starch crystal arrays. According to the plasticization theory, only the “true plasticizers” exhibited such an ability [32]. Significant lowering of the crystallinity was also observed for other acidic choline derivatives, i.e., choline lactate and acetate [15].

**Figure 5:**
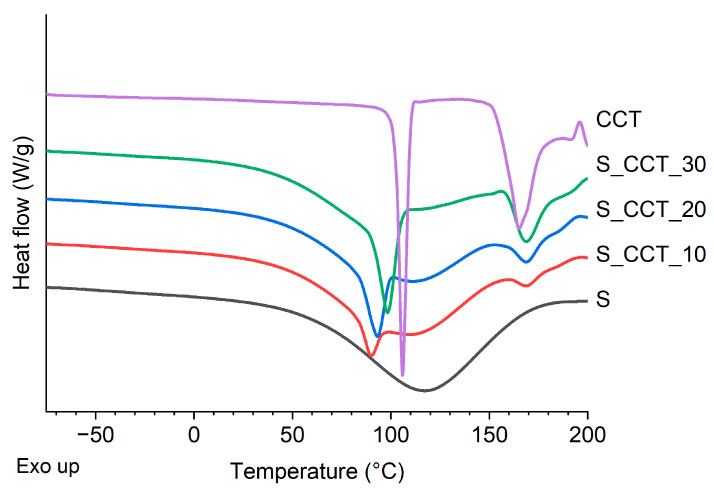
The DSC thermograms of starch-based powder mixtures with various CCT contents.

In Figure 5, the DSC thermograms of starch-based mixtures containing various CCT contents are presented. The broad endothermic transition observed on the starch thermogram is its gelatinization [34].

The sharp endothermic peak on the CCT thermogram at ca. 106 °C is the melting transition. The other endothermic transition at ca. 160 °C could be associated with the melting of citric acid followed by the CCT decomposition [35]. For the starch-based mixtures up to 20 wt.% CCT content, two transitions could be observed. The melting of CCT shifted toward lower values, e.g., to ca. 89 °C for 10 wt.% CCT, and overlapped with the starch gelatinization signal. At the same time, the starch gelatinization temperature shifted toward lower values (e.g., from ca. 115 °C to ca. 108 °C for the mixture containing 10 wt.% CCT). Interestingly, for the mixtures containing 30 wt.% CCT, only one thermal transition in this region could be observed; thus, only one phase was formed, indicating creation of the most homogeneous system and the plasticization ability of choline dihydrogencitrate salt [29].

**Figure 6:**
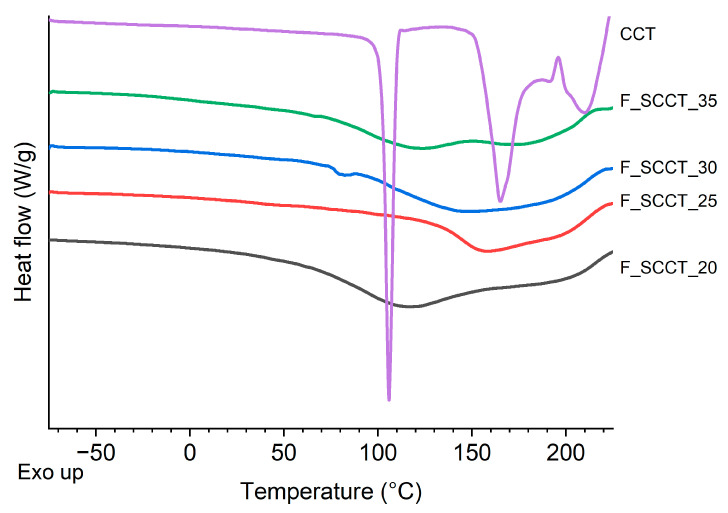
DSC thermograms of starch-based films with various CCT contents.

Interestingly, the DSC thermograms of the starch-based films containing CCT were significantly different from those of starch/CCT mixtures (Figure 6). The glass transition temperature could not be unambiguously determined from the DSC thermograms due to the absence of a well-defined heat capacity step and overlapping thermal events characteristic of starch-based materials. The thermograms were broad and exhibited overlapping endothermic transitions, suggesting the presence of multiple transition processes. Although these features may be consistent with limited compatibility or partial phase separation, DSC alone does not allow an unequivocal assessment of phase miscibility. Therefore, the films with the highest CCT content (30 and 35 wt.%) were selected for DMTA measurements to further evaluate the compatibility of the system.

**Figure 7:**
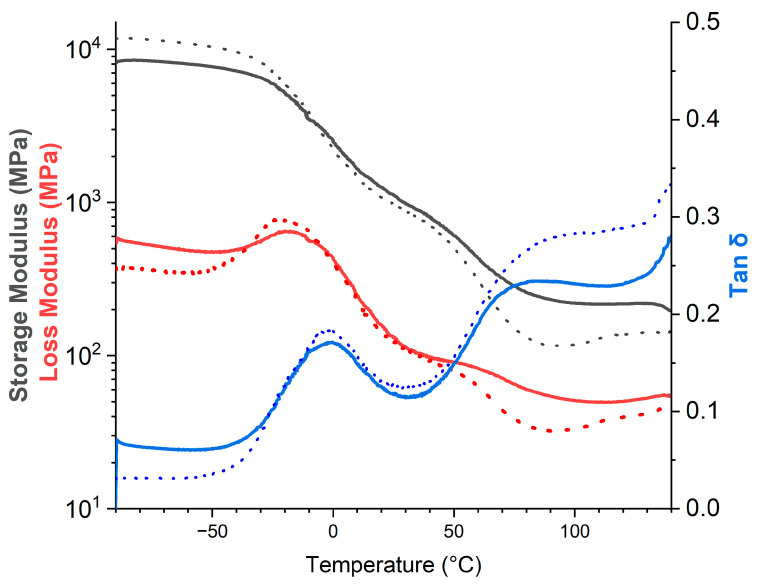
DMTA thermograms of starch-based films containing CCT 30 wt.% (solid lines) and 35 wt.% (dotted lines).

The DMTA measurements allow for evaluating the compatibility between the components. During effective starch plasticization, two main stages occur: gelatinization (plasticizer molecules irreversibly penetrate the starch granules, leading to their swelling and disruption) and fusion (the plasticizer disrupts the crystal regions of the semicrystalline polysaccharide, solvates the hydrogen bonding, and forms a homogeneous material) [28]. Tan delta and storage as well as loss modulus curves for the starch-based films containing CCT are presented in Figure 7. Two relaxation effects could be noticed: at a low (below 0 °C—domain-rich in plasticizer molecules) and at a higher temperature range (ca. 90 °C—starch-rich domains), which indicates the non-uniform distribution of plasticizer molecules in the sample volume [25]. As the peaks at tan delta and the loss modulus curves were noted at ca. 0 °C, the α-relaxation was attributed to the glass transition temperature of the plasticizer-rich phase and segmental motions of the plasticized amorphous starch phase. These motions are strongly influenced by intermolecular hydrogen bonding and the presence of plasticizer molecules. The position of this relaxation reflects the mobility of starch chains and, therefore, the efficiency of the plasticizer. For higher CCT content, a shift of α toward lower temperatures indicates enhanced chain mobility due to weakening and reorganization of intermolecular interactions. Both observations indicate the plasticizing ability of CCT toward polysaccharides [36]. 

Increasing the CCT content from 30 to 35 wt.% resulted in a reduction in the storage modulus over the entire temperature range together with a slight increase in the tan δ maximum [37]. At 25 °C, the value was in the range 0.98-1.32 GPa, and was lower for higher CCT content, suggesting more mobility of starch chains between the plasticizer molecules [38]. Similar observations were reported for thermoplastic starch plasticized with a ternary deep eutectic solvent [38] and for plasticized lignin [37].

The tan delta peak and the storage moduli decreased with higher plasticizer content. These changes indicate enhanced chain mobility and greater viscous energy dissipation, confirming the increased plasticization efficiency of CCT [37]. The relaxation at ca. 90 °C, i.e., α′-relaxation, could be attributed to starch-rich domains and some residue of starch crystals. At elevated temperature, the crystals start to transform, e.g., the melting and dissolution process could occur. Similar observations (α′-relaxation) were reported for the starch-based systems containing plasticizers with dissolving ability [25,38].

The obtained results were compared with the reports on the most common starch plasticizer, i.e., glycerol. Similarly, the glycerol-plasticized materials exhibited two phases, glycerol-rich domains with glass transitions at ca. −40 °C, and starch-rich domains with a glass transition at ca. 20 °C [39]. Both Tgs decreased with higher plasticizer content, indicating enhanced mobility of polysaccharide chains [39]. One could conclude that plasticization efficiency is governed not only by the ability of the plasticizer to form intermolecular interactions with the polymer but also by its molecular size and mobility. In contrast to glycerol, a small molecule with a melting point of approximately 18 °C, CCT possesses a larger molecular structure and a substantially higher melting point (approximately 103 °C), which are expected to influence its diffusion within the starch matrix and, consequently, the mechanism and efficiency of plasticization. 

**Figure 8:**
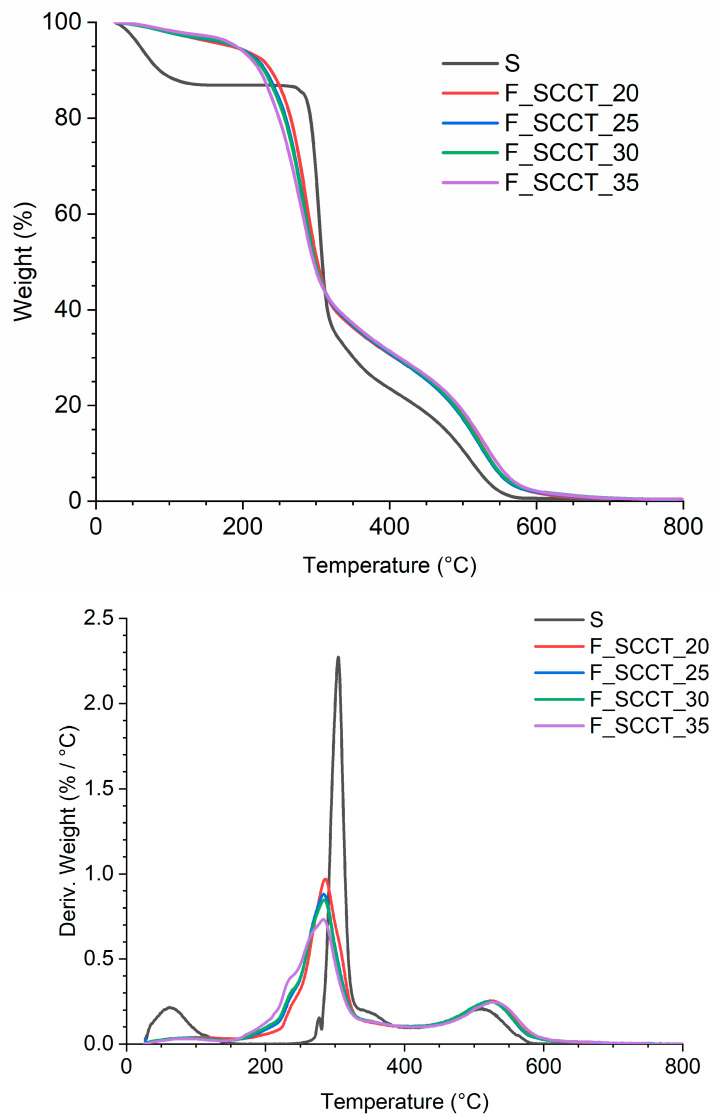
TGA and DTG thermograms of the starch-based films with various CCT contents.

Thermogravimetric analysis allows for the evaluation of the effect of plasticizer on the thermal stability of polymeric systems. In Figure 8, the TGA and DTG thermograms of the native granular starch and the starch-based films with various CCT contents were presented. Moreover, the characteristic temperatures of 5, 25, 50, and 75% mass loss were collected in Table 1. The temperature of maximum mass loss (T_max_) was determined from DTG curves.

The TGA curves of native starch showed a three-step weight loss. The first one, up to 120 °C, corresponded to the loss of moisture [38]. The main product of starch decomposition in the range 250–350 °C was water formed by condensation of hydroxyl groups [40]. The other in the range 350–550 °C referred to starch decomposition [20]. The starch-based films showed a very similar two-step weight loss, at 190–350 °C and 350–600 °C. The moisture evaporated more gradually than for native polysaccharides, indicating that water molecules were more efficiently entrapped in the starch-based structure [19]. The first decomposition stage was ascribed to dehydroxylation, and the other one to carbonization. When comparing the T5% values of the native starch and the starch-based films, the systems with CCT exhibited a higher onset temperature of degradation than the base polysaccharide. This indicated strong bonding between starch and plasticizer [41]. However, the highest value of temperature for maximum decomposition rate (DTG) was noted for native starch. It is known that natural polysaccharides exhibit higher thermal stability than the starch-based systems containing CCT (in the form of deep eutectic solvents with urea or glycerol) [20]. Moreover, it was also reported that carboxyls of CCT could form an ester bond with starch hydroxyl groups [19]. Thus, the transition and degradation of the starch/CCT films are complex, but generally, the choline dihydrogencitrate content strongly affects the thermal stability of the starch-based films.

**Figure 9:**
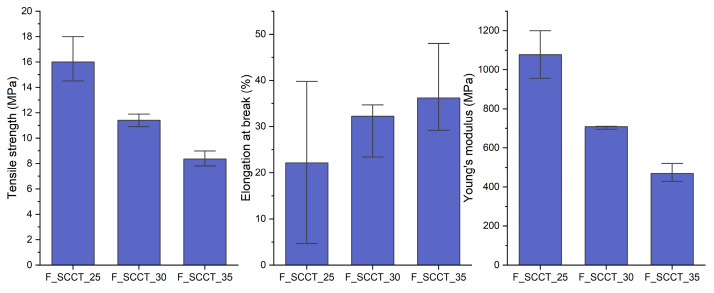
The tensile strength, elongation at break, and Young’s modulus of the starch-based films with various CCT contents.

The tensile strength, elongation at break, and Young’s modulus of the starch-based films with various CCT contents are presented in Figure 9. The high brittleness of the F_SCCT_20 film did not allow for the determination of its tensile strength. The presence of CCT in the starch-based films resulted in a reduction in both tensile strength and Young’s modulus, but the elongation at break increased. This is typical behavior of plasticizing activity [42].

An efficient plasticizer disrupts the ordered crystalline regions of starch, increasing the amorphous fraction, as evidenced by the XRD results. This structural change is consistent with the disruption of intermolecular hydrogen bonds between starch chains. However, the CCT molecules interacted with starch macromolecules, forming new H-bonding, reducing the intra-molecular interactions between polysaccharide chains and, as a consequence, increasing their mobility [43]. Simultaneously, the stretchability and flexibility of the films increased. Interestingly, it was reported that when CCT was introduced into the starch matrix in the presence of various glycerol contents, the tensile strength and elastic modulus increased. This could suggest partial chemical crosslinking of starch molecules [19]. However, the noted tensile strength values did not exceed 10 MPa, indicating that the presence of glycerol was crucial for the mechanical performance of the films. When CCT was used as the sole additive, it acted as a primary plasticizer, leading to increased flexibility rather than reinforcement of the starch matrix.

**Figure 10:**
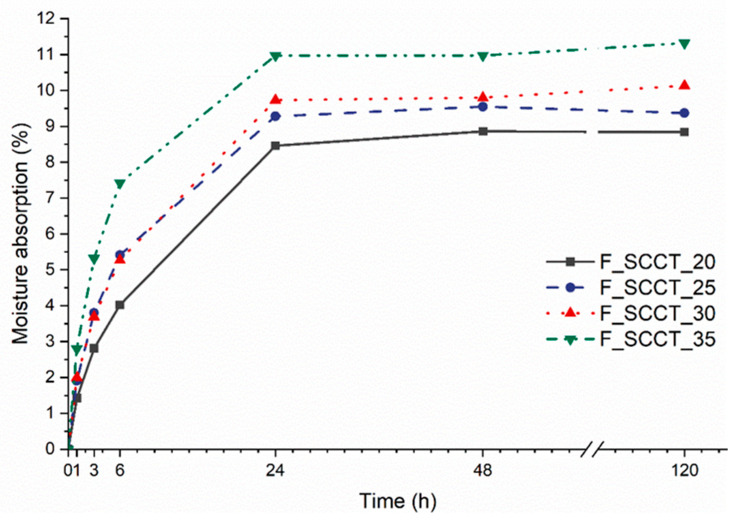
Moisture absorption for starch-based films plasticized with various CCT contents.

In Figure 10, the moisture absorption results for the starch-based films with various CCT contents are presented. Generally, the value of this parameter gradually increased with time, and after ca. 24 h, the plateau was achieved. The highly hydrophilic character of CCT strongly affected the moisture sorption, i.e., increased with plasticizer amount. Additionally, the XRD results revealed that CCT molecules could disrupt the crystal regions of the polysaccharide, and amorphous regions are more prone to retaining moisture [44]. A similar effect, i.e., moisture sorption increases with plasticizer content, was reported for other plasticizing systems based on glycerol [45], urea [25], sorbitol [46] or poly(ethylene glycol) [47]. However, when comparing the level of moisture absorption for the same plasticizer content, applying the glycerol:urea (1:1 mol) or urea:sorbitol (1:1 mol) systems resulted in at least ten times higher value (260%, and 152%, respectively) than for CCT (ca. 11% at 35 wt.% content), which makes the obtained films interesting as potentially less sensitive to moisture when stored. At comparable testing conditions (RH, temperature), the reported moisture absorption of starch plasticized with 20 wt.% of glycerol is ca. 10% [46], and for the starch-based films containing 25 wt.% of sodium citrate or poly(ethylene glycol): ca. 20% and 29% [48], respectively. For comparison, the value of this parameter for 25 wt.% CCT content is below 9%. The differences in plasticizer molecular structure could be responsible for the moisture absorption levels [46]. Although CCT contains four carboxylic and two hydroxyl groups, its molecular weight is 295 g/mol, and it is relatively bigger than, e.g., the glycerol molecule (molecular weight of 92 g/mol) or urea (60 g/mol). Thus, it showed lower capacity to interact with water [46].

**Figure 11:**
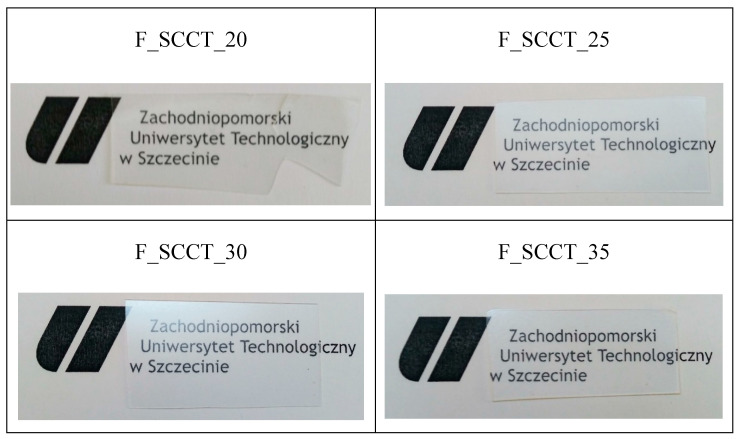
The images of the starch-based films with various CCT contents.

The photographs of the starch films with various CCT contents are presented in Figure 11. All the films were colorless, with a gloss. However, the system S_CCT_20 exhibited slightly reduced transparency when compared to the other films. The reason could be the insufficient CCT dosage, as this system exhibited the highest brittleness among the starch-based films tested.

## 4. Conclusions

For the first time, choline dihydrogencitrate salt, which is a metabolic precursor to vital bodily compounds, was tested as a sole plasticizer for starch-based materials. The effect of CCT content on the physicochemical properties of the obtained films was evaluated. The XRD measurements revealed that CCT exhibited the ability to disrupt the crystalline regions of potato starch. Also, DMTA and the mechanical test results showed that this environmentally friendly ionic liquid acted as a primary plasticizer, leading to increased flexibility of starch-based films. The efficient CCT content, considering transparency, was at least 25 wt.%. However, on the other side, increasing the CCT content in the starch-based systems resulted in lowertensile strength and elasticity of the films. The onset temperature of degradation was higher for the materials with CCT addition than for neat starch. Interestingly, the obtained starch/CCT materials exhibited lower moisture absorption levels than those with other common starch plasticizers.

## Figures and Tables

**Table 1:** TGA temperatures of 5%, 25%, 50%, and 75% weight loss, and T_max_.

Sample	T_5%_	T_25%_	T_50%_	T_75%_	T_max_
	[°C]	[°C]	[°C]	[°C]	[°C]
S	60	296	308	386	304
F_SCCT_20	185	273	302	455	285
F_SCCT_25	189	267	298	453	284
F_SCCT_30	190	265	298	456	284
F_SCCT_35	191	259	296	460	283

## Data Availability

The data presented in this study are available on request from the corresponding author due to privacy.

## Supplementary Materials

The following supporting information can be downloaded at: https://www.mdpi.com/article/10.3390/ma19153242/s1, Figure S1: The FTIR spectra of CCT, and starch-based film with various CCT content in (A) full wavenumber range, (B) in the wavenumber range of 1500–1800 cm^−1^, and (C) in the wavenumber range of 900–1200 cm^−1^.
