# Supplementary material for: The Plasticizing Effect of Choline Dihydrogencitrate on Starch-Based Films
Source: Materials (Basel). 2026 Jul 31;19(15):3242. doi: 10.3390/ma19153242 (PMC13466891; doi:10.3390/ma19153242)
Supplement: Supplementary file 1 [file materials-19-03242-s001.zip › materials-4424425-supplementary.pdf]

The plasticizing effect of choline dihydrogencitrate on starch-based films

Dorota Skowrońska[1], Katarzyna Wilpiszewska[2]*

West Pomeranian University of Technology, Faculty of Chemical Technology and Engineering, Department of Chemical Organic Technology and Polymeric Materials, ul. Pulaskiego 10, 70-322 Szczecin, Poland

[1]Email D. Skowrońska: Dorota.Skowronska@zut.edu.pl

[2]*Corresponding author. E-mail: kwilpi@zut.edu.pl (K. Wilpiszewska)

Tel. +48 91 449 41 78

Initially, it was planned to investigate starch-based systems containing 10–35 wt.% CCT. However, the films with low CCT content, i.e. below 20 wt.% exhibited significant brittleness and therefore could not be subjected to mechanical testing. The films could not be removed from the moulds as intact specimens because they fractured immediately during demoulding. For the same reason, DMTA measurements could not be performed, as this technique requires intact specimens of defined geometry that can be properly mounted and tested in several replicates. XRD analyses were also not performed for the formulations containing 10 and 15 wt.% CCT because sufficiently large, flat, and representative film fragments suitable for the measurement configuration used in this study could not be obtained. Importantly, the other formulations were analysed by XRD in the form of films; therefore, testing the brittle samples in a powdered or fragmented form would have introduced differences in sample preparation and reduced the direct comparability of the results. However, FTIR (ATR) analyses were performed for the formulations containing 10 and 15 wt.% CCT, as this

technique requires only a small sample fragment, and cracking of the film does not prevent measurement provided that adequate contact with the ATR crystal is achieved.

In Fig. 1S the FTIR spectra of CCT, and starch-based filme with the various CCT content: 10-35 wt.% was presented.

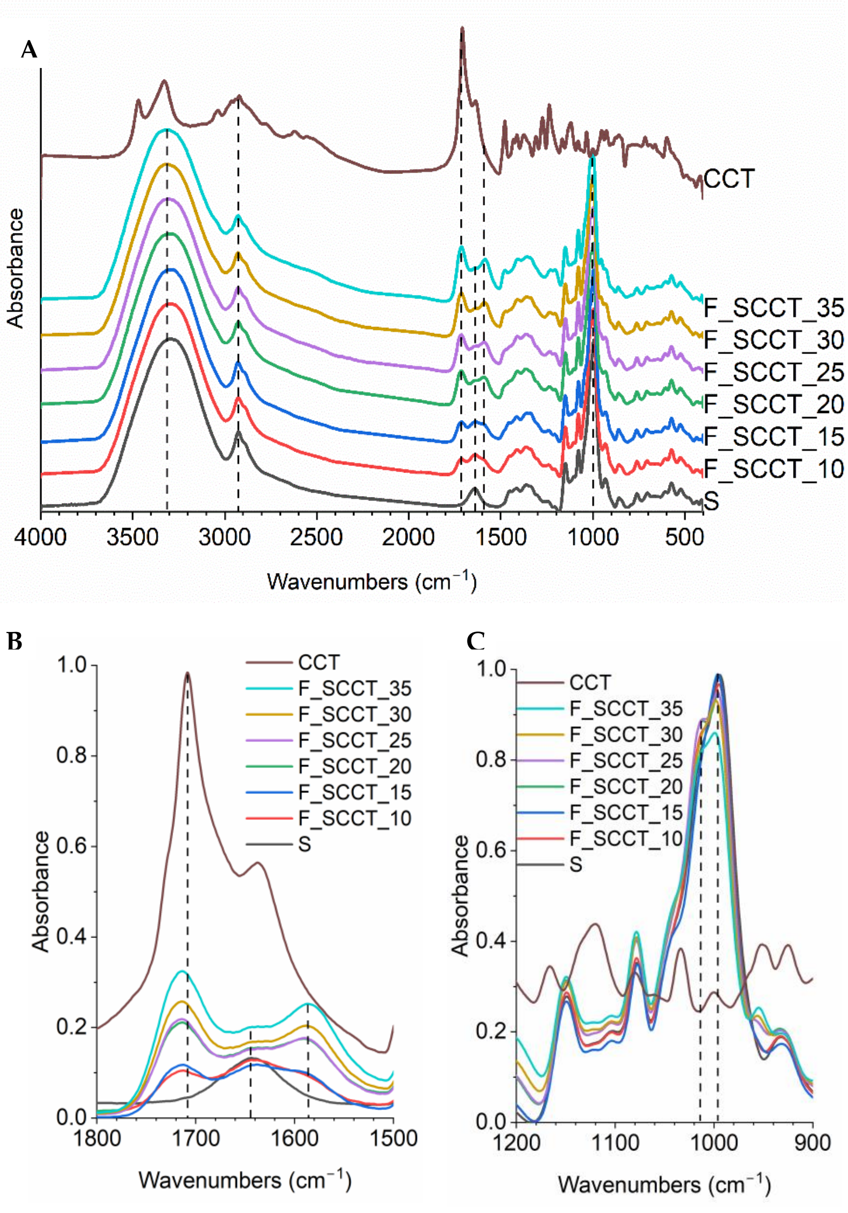

Fig. S1. The FTIR spectra of CCT, and starch-based film with various CCT content in (A) full wavenumber range, (B) in the wavenumber range of 1500-1800 cm $^{-1}$, and (C) in the wavenumber range of 900-1200 cm $^{-1}$
